# Chemical fertilizer reduction combined with bio-organic fertilizers increases cauliflower yield *via* regulation of soil biochemical properties and bacterial communities in Northwest China

**DOI:** 10.3389/fmicb.2022.922149

**Published:** 2022-07-27

**Authors:** Xuemei Xiao, Ju Li, Jian Lyu, Zhi Feng, Guobin Zhang, Haixing Yang, Chengfei Gao, Li Jin, Jihua Yu

**Affiliations:** ^1^College of Horticulture, Gansu Agricultural University, Lanzhou, China; ^2^State Key Laboratory of Aridland Crop Science, Gansu Agricultural University, Lanzhou, China; ^3^Agricultural Technology Extension Center of Yuzhong County, Lanzhou, China

**Keywords:** yield, soil property, bacterial community, bio-organic fertilizer, cauliflower

## Abstract

The continuous application of chemical fertilizers in vegetable cropping has led to deterioration of the soil environment and reduced yield and quality. The objective of this study was to evaluate the effect of combining chemical and bio-organic fertilizers on cauliflower yield, soil biochemical properties, and the bacterial community. Six treatments were established: no fertilizer (CK, control), chemical fertilizers (CF, conventional dosage for this region), balanced fertilization (BF, 30% reduction of chemical fertilizers), and balanced fertilization plus 3,000, 6,000, or 12,000 kg.ha^–1^ bio-organic fertilizer (Lvneng Ruiqi Biotechnology Co., Ltd., Gansu, China) (BF + OF1, BF + OF2, BF + OF3, respectively). A two-season field experiment with cauliflower was conducted under the different fertilizer treatments in irrigation districts along the Yellow River, Northwest China. The results indicate that the yield, soil organic matter, total potassium content, and enzyme activity under the bio-organic treatments were generally higher than those under the CF treatment. Compared with the CF treatment, the BF treatment increased soil organic matter content, enzyme activity and soil bacterial relative abundance. Moreover, the bacterial alpha-diversity were higher than those of conventional fertilization. The predominant phyla, including Proteobacteria, Actinobacteria, Gemmatimonadetes, and Chloroflexi, were the main contributors to the microbiome shift, as demonstrated by their remarkable enrichment in the soil under BF + OF2 and BF + OF3 treatments. Furthermore, Pearson correlation analyses show significant correlations among the soil organic matter, available P and K, electrical conductivity, and relative abundance of potentially beneficial microbial groups, such as the genera *Massilia, Bacillus*, *Lysobacter*, and *Nitrosospira*. Overall, this study suggests that balanced fertilization and the application of bio-organic fertilizers are essential to ensure soil fertility and long-term sustainable green productivity.

## Introduction

Vegetables are among the foods with the largest per capita consumption globally. Moreover, vegetable production has become one of the primary sources of per capita income growth among rural residents worldwide (Gai et al., 2016). The large rise in chemical fertilizer inputs has resulted in a tremendous boost in vegetable yield in China during the last 20 years (Dawson and Hilton, 2012; Souza et al., 2020). However, chemical fertilizer application also increased production cost and led to the misuse of fertilizers, disruption to the stability of the soil environment, and decreased the quality of vegetables (Wang et al., 2017; Wesenbeeck et al., 2021; Yin et al., 2021). Recently, concerns have been raised about planting pattern productivity and sustainability, which has remained stagnant or even declined. Therefore, coordinating the relationship between chemical fertilizer input and the high-stable yield of vegetables is a problem that urgently needs to be solved in current agricultural vegetable production.

Balanced fertilization and the combined application of organic and chemical fertilizers are beneficial for the sustainable development of vegetable production (Zhao et al., 2016; Wang et al., 2021). Balanced fertilization technology was designed according to the nutrient requirements of specific crops during growth and fruiting. In addition, the effects of climate and environmental factors on crop nutrient absorption were considered (Singh et al., 2015). Balanced fertilization can increase crop yield, create scientific, standardized, and accurate fertilization management, and reduces fertilizer waste (Lin et al., 2012). Bio-organic fertilizer is a compound fertilizer that uses animal and plant residues as raw material, which is produced by adding specific functional microorganisms and harmless decomposed organic substances (Naher et al., 2021). The organic matter content is very high (≥40%), which improves soil quality and enhances the nutrient supply capacity (Aparna et al., 2014; Qiao et al., 2019). Soil organic matter provides all the nutrients needed for crop growth, and prevents nutrient leaching. Soil microorganisms play an indispensable role in terrestrial ecosystems, they are involved in producing and transforming organic matter in the soil, nutrient cycling, and improving the utilization efficiency of nutrients by crops (Paz-Ferreiro et al., 2014; Lin et al., 2016; Paul, 2016). Previous studies have shown that balanced fertilization and the combined application of bio-organic fertilizers can improve crop yield, possibly owing to the alteration of soil properties, enzyme activities, and soil microorganisms. Yu et al. (2019) and Brenzinger et al. (2021) found that balanced fertilization could improve soil fertility by increasing soil cation exchange capacity (ECE), soil organic matter (SOM), and available phosphorus (AP) content as well as transforming soil microorganisms. By combining bio-organic fertilizers with chemical fertilizers, organic matter in the soil can be increased and soil enzymes and microorganisms can be stimulated (Baldi et al., 2016; Jiang et al., 2019).

Cauliflower (*Brassica oleracea var. botrytis* L.) is one of the main plateau summer vegetables crops in the Yellow River irrigation area of Northwest China. However, there is concern regarding the problem of unbalanced fertilization during cauliflower production. Excessive chemical fertilization leads to low fertilizer use efficiency and yield in cauliflower cropping (Yang et al., 2020). We speculated that balanced fertilization and application of bio-organic fertilizer could improve fertilizer use efficiency and yield of cauliflower by altering the soil biochemical environment. Therefore, we a field experiment was conducted on cauliflower with reduced chemical fertilizers and combination of the later with bio-organic fertilizers. To investigate how fertilizers fertilization affect vegetable cauliflower yields and, soil enzyme activity, and composition and diversity of microbial community by high-throughput sequencing. The results are expected to provide insights to increase fertilizer use efficiency, reduce fertilizer input, and offer a reference for developing effective fertilizer management strategies for vegetable production.

## Materials and methods

### Site and soil description

The experimental site was one of main plateau summer vegetables production areas in Lanzhou City, Gansu Province, China (35°87′N, 104°23′E). The region has a temperate semi-arid continental climate with distinct seasons, mean altitude is 1,790 m above sea level, mean annual temperature of 6.6^°^C, mean annual rainfall of 300–400 mm, mean annual evaporation of 1,343 mm, and a frost-free period of approximately 150 days. The test field has gentle terrain and the fertility level was uniform. The soil is a typical loessal, and the main physicochemical parameters of the 0–20 cm topsoil were pH 7.85, SOM 12.08 g.kg^–1^, total nitrogen (TN) 1.01 g.kg^–1^, AP 97.66 mg.kg^–1^, and available potassium (AK) 138.44 mg.kg^–1^.

### Experimental design

The field experiment was performed in a completely randomized block design with three replicates for each of the following treatments: CK, no fertilizer (control); CF, chemical fertilizers (conventional dosage for this region, N 805.5 kg.ha^–1^, P_2_O_5_ 948 kg.ha^–1^, K_2_O 420 kg.ha^–1^); BF, balanced fertilizer (30% reduction of chemical fertilizers); BF + OF1, BF + OF2, and BF + OF3, balanced fertilizer plus 3,000, 6,000, or 12,000 kg.ha^–1^ bio-organic fertilizer, respectively. The bio-organic fertilizer (Lvneng Ruiqi Biotechnology Co., Ltd., Gansu, China) was N + P_2_O_5_ + K_2_O ≥ 5%; it contained > 40% organic matter, >25% of humic acid, and >20 million effective living bacteria per gram.

The cauliflower cultivation mode was adopted for two seasons per year. For the spring crop, bio-organic fertilizer, phosphorus, potassium, and 30% nitrogen were used as the base fertilizer and applied once before planting and mixed thoroughly. The remaining 70% nitrogen fertilizer was used as topdressing at the rosette stage (50%) and early flowering stage (20%). In the autumn cultivation, the same fertilizer types and methods were used as controls, but reduced by 30%, and the NPK ratio was adjusted. All fertilizers were applied three times at the rosette stage, the early stage of curd formation, and the peak stage of curd formation. The fertilization amounts for each treatment are summarized in Table 1.

**TABLE 1:** Fertilizer amounts for different treatments.

Treatment	Bio-organic fertilizer (kg.ha^–1^)	Spring crop (kg.ha^–1^)			Autumn crop (kg.ha^–1^)			Total (kg.ha^–^^1^)
		N	P_2_O_5_	K_2_O	N	P_2_O_5_	K_2_O	
CK	0	0	0	0	0	0	0	0
CF	0	513	573	276	292.5	375	144	2173.5
BF	0	405	148.5	399	315	93	160.5	1,521
BF + OF1	3,000	405	148.5	399	315	93	160.5	1,521
BF + OF2	6,000	405	148.5	399	315	93	160.5	1,521
BF + OF3	12,000	405	148.5	399	315	93	160.5	1,521

CK, no fertilizer; CF, chemical fertilizers (conventional dosage for this region, the total amount of nitrogen, phosphorus and potassium fertilizer in two seasons of a year is: N 805.5 kg.ha^–1^, P_2_O_5_ 948 kg.ha^–1^, K_2_O 420 kg.ha^–1^); BF, balanced fertilizer (30% reduction of chemical fertilizers, the total amount of chemical fertilizer is reduced by 30% compared with the CF treatment. After adjusting according to the fertilizer demand of crops, the application of N is reduced by 10.6%, the application of P_2_O_5_ is reduced by 74.5%, and the application of K_2_O is increased by 33.2%.); BF + OF1, BF + OF2, and BF + OF3, balanced fertilizer plus 3,000, 6,000, and 12,000 kg.ha^–1^ bio-organic fertilizer, respectively (the total amount of chemical fertilizer is reduced by 30% compared with the CF treatment and combined with bio-organic).

### Cauliflower yield and economic benefit

Each treatment had three trial plots, each with an area of 31.5 m^2^ (4.5 × 7 m). When the cauliflower met the harvest standard, the middle section of each plot of 18 m^2^ (3 × 6 m) was selected; the economic yield (wet weight of curd) within this section was measured and converted into yield per hectares based on the yield per unit area.

Economic benefit (yuan.667 m^–2^) = Annual total output (yuan.667 m^–2^) − Total fertilizer input (yuan.667 m^–2^).

### Soil sample collection

A soil sample was taken at a depth of 0–20 cm after removal of 1 cm deep topsoil during the harvest period of autumn cauliflower. Three parallel samples were collected using a five-spot sampling method for each treatment. We collected soil samples and immediately loaded them into ice boxes and transported them to the laboratory within 1.5 h. The soil from the 15 points in each treatment was thoroughly mixed to remove stones, roots, plastic film debris, and other impurities, and then passed through a sieve of 2 mm in size. We divided each sample into two parts: one part was frozen at −80^°^C for later extraction of soil DNA. In a laboratory environment, the remaining soil was placed in a ventilated area and dried so that it reached a constant weight, after which its physical and chemical properties and enzyme activities were determined.

### Soil chemical property and enzyme activity analysis

The pH of the soil samples was measured using a pH electrode (PHS-3C, Shanghai Yidian Scientific Instrument Co., Ltd., Shanghai, China) at a soil/water ratio of 1:5. The electrical conductivity (EC) of the soil samples was measured using a conductivity meter (DDS-307A, Shanghai Yidian Scientific Instrument Co., Ltd., Shanghai, China) at a soil/water ratio of 1:5. The potassium dichromate volumetric method was used to measure the SOM content. The TN, total phosphorus (TP), and total potassium (TK) contents were measured after the digestion with H_2_SO_4_-H_2_O_2_ mixture. TN content was analyzed using a Kjeldahl nitrogen analyzer, TP content was measured using the Mo-Sb colorimetric method, and TK content was determined using flame spectrometry. Alkali-hydrolyzed nitrogen (Alk N), AP, and AK contents were analyzed using diffusion, Olsen, and ammonium acetate extraction flame photometry methods, respectively. These methods were modified according to Lu (1999). Soil enzyme (sucrase, urease, and alkaline phosphatase) activity was estimated by colorimetry (Guan et al., 1986). The soil enzyme activities of β-glucosidase and cellulase were measured using a soil β-glucosidase (S-β-GC) activity assay kit (BC0165) and soil cellulase (S-CL) activity assay kit (BC0155, Solarbio Biotech Co., Ltd., Beijing, China), respectively. To guarantee the validity of the experimental data, three independent tests were performed on each sample.

### DNA extraction and high-throughput sequencing

A 16S rDNA amplicon sequencing was carried out by MiSeq at Novogene Biotechnology Co., Ltd. (Beijing, China). Genomic DNA was extracted from the samples using thecetyltrimethylammonium bromide (CTAB) method. The DNA samples were diluted to 1 ng.μL^–1^ with sterile water. The 16S rRNA genes of distinct regions (16S V3-V4) were amplified using a specific primer [16S V4: 515F (5′-GTGCCAGCMGCCGCGGTAA-3′) −806R (5′-GGACTACHVGGGTWTCTAAT-3′)] with the barcode (Caporaso et al., 2011; Apprill et al., 2015; Parada et al., 2016). All PCRs were carried out with 15 μL of Phusion^®^ High-Fidelity PCR Master Mix (New England Biolabs, Beijing, China), 0.2 μM of forward and reverse primers, and ∼10 ng template DNA. The thermal cycling procedure included initial denaturation at 98^°^C for 1 min followed by 30 cycles of denaturation at 98^°^C for 10 s, annealing at 50^°^C for 30 s, and elongation at 72^°^C for 30 s. The PCR products were then purified. The TruSeq^®^ DNA PCR-free sample preparation kit was used to construct a library. The library quality was assessed on the Qubit@ 2.0 Fluorometer (Thermo Scientific) and Agilent Bioanalyzer 2100 system. After the library had been quantified, it was sequenced using a NovaSeq6000.

### Analysis of the sequencing data

According to the barcode sequence and PCR primer sequences, each sample was separated from the offline data. After the barcode and primer sequences were truncated, FLASH (V1.2.7)^1^ (Magoc and Salzberg, 2011) was used to splice the reads of each sample, and the spliced sequence was counted as the original tags data (raw tags). In order to obtain high-quality tags (clean tags) from spliced raw tags, strict filtering is required (Bokulich et al., 2013). The tag quality control process of Qiime (V1.9.1)^2^ (Caporaso et al., 2010) was used with the following operations: (a) tag interception: raw tags were truncated at the first low-quality base site when the continuous low-quality value (default quality threshold ≤19) was set to a default length (default length 3); (b) tag length filtering: the tag dataset obtained by interception of tags further filtered out tags with continuous high-quality base lengths of <75% of tag length. Following the above processing, the tags were further processed to remove the chimera sequence^3^ by comparison with the species annotation database to produce the final effective data (effective tags) (Rognes et al., 2016).

### Statistical analysis

One-way analysis of variance (ANOVA) with Duncan’s honestly significant difference (HSD) test (*P* < 0.05) was conducted using the SPSS software to analyze the data for each set of physicochemical factors and enzyme activity indices. The Uparse software (Uparse v7.0.1001)^4^ was used to cluster all effective tags of all the samples (Haas et al., 2011). Based on 97% identity, the sequences were clustered into operational taxonomic units (OTUs) and representative sequences of OTUs were selected. In accordance with the algorithm principle, the sequences with the highest frequency within the OTUs were selected as representative sequences within the OTUs. The species annotation analysis was carried out by using the Mathur method and the SSUrRNA database of SILVA132^5^ (Wang, 2007; Edgar, 2013). The α-Diversity indices (including goods coverage, PD_whole_tree index, Chao1, Shannon, Simpson, and ACE) were calculated using the Qiime software (version 1.9.1). The Unifrac distance was computed using the Qiime software (version 1.9.1), and the principal component analysis (PCA) diagram was drawn using R (version 2.15.3). PCA for OTUs (97% similarity) can reflect the differences and distances among samples. The higher the similarity between two samples, the closer the distance between them. The similarity of the sample composition under the same conditions can be judged according to the dispersion and aggregation of the samples. Using the corr.test function of the Psych package in R, we calculated the Spearman correlation coefficient between species and environmental factors and tested the significance. To visualize the heatmap, the pheatmap function was used in the “pheatmap” package.

## Results

### Cauliflower yield and economic benefits under different fertilization treatments

The various fertilizer applications strongly influenced the cauliflower yield (Table 2). The BF + OF2 and BF + OF3 treatments significantly increased cauliflower yield by 7.5 and 5.9% compared to CF, respectively in the spring crop. In autumn crops, the yield difference between BF and CF treatments was not significant. The highest yield was achieved by the BF + OF3 treatment, which was 18.6% higher than that of the CF treatment. The second largest total yield was observed in the BF + OF2 treatment, which increased yield by 11.3% compared with that of the CF treatment. Overall, the treatments with balanced fertilizer plus bio-organic fertilizer produced higher yields than the treatments with only chemical fertilizer (CF) or balanced fertilizer (BF). Compared with CK2, the economic benefits of T2, T3 and T4 treatment increased by 5.8, 6.8, and 6.1%, respectively. The fertilizer cost of T3 treatment increased by 320 yuan.667 m^–2^ and the economic benefits increased by 190.9 yuan.667 m^–2^, compared to T2 treatment. The fertilizer cost of T4 treatment increased by 640 yuan.667 m^–2^ compared with T3 treatment, but the economic benefit decreased. Therefore, the economic benefit of T3 treatment was the best, which was 19777.0 yuan.667 m^–2^, increased by 1265.5 yuan.667 m^–2^ compared with CK2 treatment.

**TABLE 2:** Cauliflower yield under different fertilization treatments.

Treatment	Spring crop		Autumn crop		Annual Total output (yuan.667 m^–2^)	Annual fertilizer inputs (yuan.667 m^–2^)	Annual economic benefits (yuan.667 m^–2^)
	Yield (kg.667 m^–2^)	Yield increase rate%	Yield (kg.667 m^–2^)	Yield increase rate%			
CK	2832.4 ± 35.2c	–	2487.9 ± 65.5d	–	17093.86	0	17093.86
CF	3044.1 ± 63.5b	–	3021.9 ± 87.8c	–	19415.64	904.1	18511.54
BF	3154.8 ± 49.1ab	3.6	2909.7 ± 71.5c	–	19455.42	793.3	18662.12
BF + OF1	3172.5 ± 48.3ab	4.2	3304.3 ± 95.4b	9.3	20699.4	1113.3	19586.10
BF + OF2	3271.5 ± 63.2a	7.5	3362.4 ± 63.0ab	11.3	21210.3	1433.3	19777.00
BF + OF3	3223.0 ± 63.1a	5.9	3584.7 ± 102.8a	18.6	21712.3	2073.3	19639.00

CK, no fertilizer; CF, chemical fertilizers (conventional dosage for this region); BF, balanced fertilizer (30% reduction of chemical fertilizers); BF + OF1, BF + OF2, and BF + OF3, balanced fertilizer plus 3,000, 6,000, and 12,000 kg.ha^–1^ bio-organic fertilizer, respectively. Data are expressed as the mean ± standard error (SE) of three replicates. Different letters indicate significant differences at p < 0.05, according to Duncan’s test. Urea 2 yuan . kg^–1^, calcium superphosphate 1 yuan . kg^–1^, potassium sulfate 6 yuan . kg^–1^, diammonium phosphate 0.9 yuan . kg^–1^, compound fertilizer 1.6 yuan . kg^–1^, nitrate sulfur base 103 4.6 yuan . kg^–1^, ammonium calcium nitrate 2 yuan . kg^–1^, bio-organic fertilizer 1.6 yuan . kg^–1^, spring and Autumn Cauliflower 3.4 and 3.0 yuan . kg^–1^, respectively.

### Soil properties under different fertilization treatments

Compared with the conventional CF treatment, balanced fertilization combined with biological organic fertilizers increased the soil nutrient content, except for TP, and decreased the pH and EC (Table 3). Compared with that of CF treatment, soil organic matter content in the BF + OF1, BF + OF2, and BF + OF3 treatments increased by 2.0, 2.1, and 3.7%, respectively. The TP content decreased by 10.0 ∼16.9%. The soil TN and TK contents were not significantly different under the different fertilization treatments. The soil AK content in the BF + OF3 treatment was markedly higher than that in the CF treatment, and increased by 20.1%, while the AP contents in the BF, BF + OF1, BF + OF2, and BF + OF3 treatments were significantly lower than that of the CF treatment (decreases of 30.0∼61.3%). The soil pH of the BF + OF2 and BF + OF3 treatments were decreased by 1.1 and 2.5%, respectively, compared with that of the CF treatment. The EC values decreases by 6.4 ∼13.2% in the BF + OF1, BF + OF2, and BF + OF3 treatments compared with the CF treatment.

**TABLE 3:** Soil available nutrients and organic matter under different fertilization treatments.

Treatment	SOM (g.kg^–1^)	TN (g.kg^–^^1^)	TP (g.kg^–^^1^)	TK (g.kg^–^^1^)	AIK N (mg.kg^–^^1^)	AP (mg.kg^–^^1^)	AK (g.kg^–^^1^)	pH	EC (us.cm^–^^1^)
CK	11.49 ± 0.43b	0.51 ± 0.03c	1.01 ± 0.01de	7.23 ± 0.12c	45.03 ± 1.23c	43.46 ± 0.13d	63.33 ± 0.88e	7.14 ± 0.03b	458.00 ± 2.52d
CF	13.26 ± 0.71ab	0.76 ± 0.06ab	1.30 ± 0.02a	7.90 ± 0.10ab	60.90 ± 2.14ab	119.09 ± 4.24a	139.33 ± 2.67b	7.21 ± 0.01a	593.00 ± 5.69a
BF	12.10 ± 0.27ab	0.62 ± 0.03bc	0.96 ± 0.03e	7.55 ± 0.09bc	45.97 ± 2.60c	46.13 ± 1.84d	92.67 ± 1.86d	7.13 ± 0.01b	431.33 ± 8.33e
BF + OF1	13.53 ± 0.81a	0.65 ± 0.04bc	1.16 ± 0.03bc	7.97 ± 0.09a	55.77 ± 1.23b	74.09 ± 1.11c	122.67 ± 2.73c	7.16 ± 0.01ab	514.67 ± 5.70c
BF + OF2	13.69 ± 0.15a	0.71 ± 0.04ab	1.08 ± 0.01cd	8.10 ± 0.16a	62.30 ± 1.40a	79.67 ± 2.73bc	144.67 ± 2.19b	7.13 ± 0.01b	543.33 ± 6.39b
BF + OF3	13.75 ± 0.64a	0.84 ± 0.05a	1.17 ± 0.04b	8.10 ± 0.10a	63.23 ± 2.47a	83.41 ± 1.40b	167.33 ± 1.86a	7.03 ± 0.03c	555.33 ± 7.62b

CK, no fertilizer; CF, chemical fertilizers (conventional dosage for this region); BF, balanced fertilizer (30% reduction of chemical fertilizers); BF + OF1, BF + OF2, and BF + OF3, balanced fertilizer plus 3,000, 6,000, 12,000 kg.ha^–1^ bio-organic fertilizer, respectively. Data are expressed as the mean ± standard error (SE) of three replicates. Different letters indicate significant differences at p < 0.05, according to Duncan’s test. SOM, soil organic matter; TN, Total nitrogen; TP, total phosphorus; TK, total potassium; AIK, available nitrogen; AP, available phosphorus; AK, available potassium.

### Soil enzyme activities under different fertilization treatments

**FIGURE 1:**
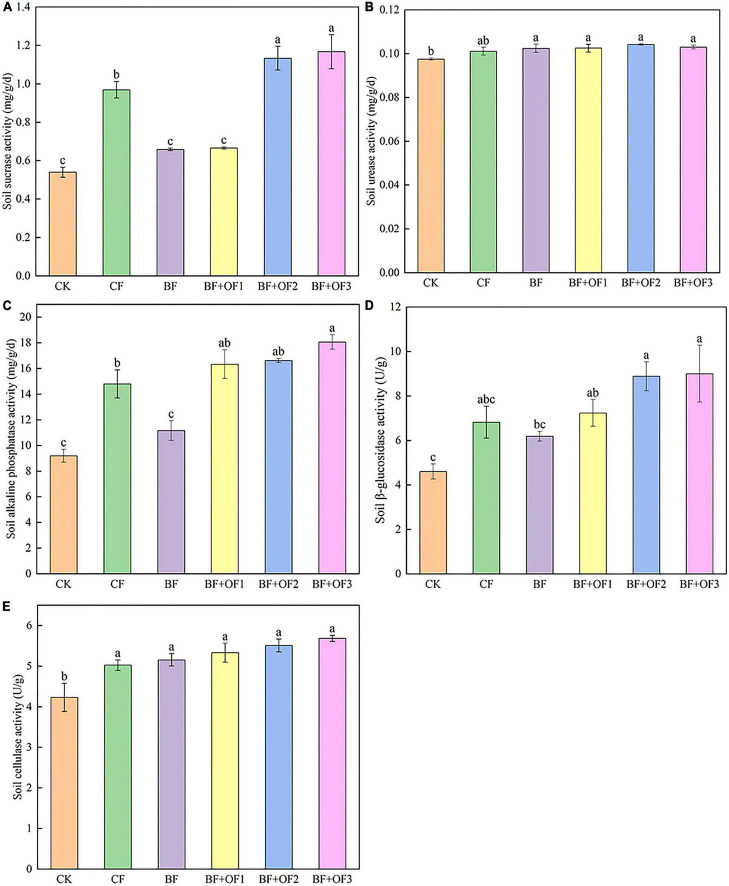
Soil enzyme activities under different fertilization treatments. The sucrase activities **(A)**, urease activities **(B)**, alkaline phosphatase activities **(C)**, β-glucosidase activities **(D)** and cellulase activity **(E)**. CK, no fertilizer, CF, chemical fertilizers (conventional dosage for this region); BF, balanced fertilizer (70% of total chemical fertilizer); BF + OF1, BF + OF2, and BF + OF3, balanced fertilizer plus 3,000, 6,000, and 12,000 kg⋅ha^–1^ bio-organic fertilizer, respectively. Each histogram represents the mean ± standard error (SE) of three independent biological experiments (*n* = 3). Different letters above the bars indicate statistically significant differences by Duncan’s test (*p* < 0.05).

The soil enzyme activities of the different fertilization treatments are shown in Figure 1. Balanced fertilization and bio-organic fertilizers improved soil enzyme activity. Compared with that of the CF treatment, the sucrase activities of the BF + OF2 and BF + OF3 treatments increased by 17.0 and 20.4%, respectively (Figure 1A). The urease activities of the BF, BF + OF1, BF + OF2, and BF + OF3 treatments increased by 1.3, 1.4, 3.1, and 1.9%, respectively, compared with that of the CF treatment (Figure 1B). The activity of alkaline phosphatase in the CK treatment was the lowest. The alkaline phosphatase activities in the BF + OF1, BF + OF2, and BF + OF3 treatments were higher than that in the CF treatment. Among them, the BF + OF3 treatment reached a significant difference (*P* < 0.05), increasing the alkaline phosphatase activity by 22.1% (Figure 1C). The order of β-glucosidase activity was BF + OF3 > BF + OF2 > BF + OF1 > CF > BF > CK in six soil treatment. The β-glucosidase activities of BF + OF1, BF + OF2, and BF + OF3 treatment increased by 6.1, 30.2, and 32.0%, respectively (Figure 1D). Compared with that of the CF treatment, the BF, BF + OF1, BF + OF2, and BF + OF3 treatments increased soil cellulase activity by 2.6, 6.1, 9.7, and 13.2%, respectively (Figure 1E).

### Soil bacterial community structure under different fertilizer treatments

#### Soil bacterial α-diversity under different fertilization treatments

**FIGURE 2:**
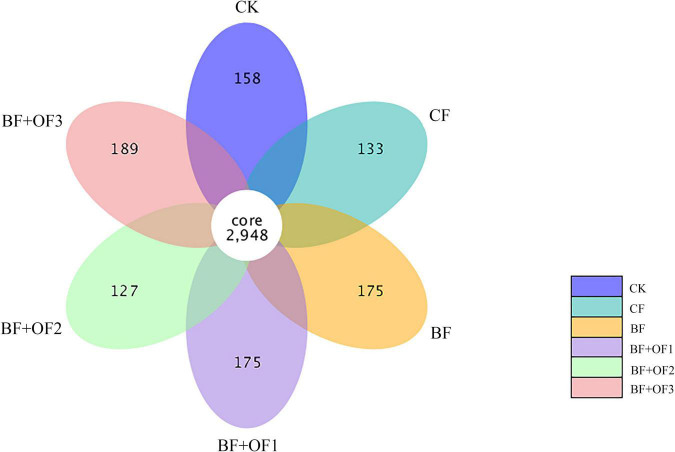
Petal plot of soils with different fertilization treatments based on operational taxonomic unit (OTU) distribution. CK, no fertilizer, CF, chemical fertilizers (conventional dosage for this region); BF, balanced fertilizer (70% of total chemical fertilizer); BF + OF1, BF + OF2, and BF + OF3, balanced fertilizer plus 3,000, 6,000, 12,000 kg.ha^–1^ bio-organic fertilizer, respectively.

The common number of OTUs in the six treatments are 2,948, and the number of unique OTUs in each treatment was 158 (CK), 131 (CF), 175 (BF), 175 (BF + OF1), 127 (BF + OF2), and 189 (BF + OF3) (Figure 2). Generally, the number of unique OTUs in the chemical fertilizer reduction combined with bio-organic fertilizers increased compared with that of the CF treatment. The number of unique OTUs in BF and BF + OF1 increased by 44%, while those in BF + OF3 increased by 58% compared with that of the CF treatment.

Table 4 shows that the sequencing data can effectively express almost 99% of the bacteria in soil samples and cover almost all possible species. Fertilizer reduction based on Balanced Fertilization did not reduce the diversity of soil microorganisms, and there was no significant difference in Chao1 and ACE indexes among fertilization treatments. Compared with the CF treatment, the bacterial Shannon indices of BF, BF + OF1, and BF + OF3 treatments increased to different degrees. The Shannon index of the BF + OF3 was the highest. The greater the Simpson index value, the lower the community diversity. In general, the Simpson index difference between treatments was not significant. The Simpson index of The BF and BF + OF1 treatment are slightly lower than of CF treatment. The results show that chemical fertilizer reduction combined with bio-organic fertilizers could improve the diversity of soil bacteria to a certain extent.

**TABLE 4:** Species number and bacterial diversity index under different treatments.

Treatment	Observed_species	Chao1	ACE	Shannon	Simpson	PD_whole_tree	Coverage %
CK	3523.7 ± 26.3ab	3955.0 ± 69.9a	4018.9 ± 66.6ab	9.78 ± 0.03a	0.996 ± 0.000a	234.5 ± 3.5ab	98.7
CF	3508.0 ± 44.8ab	3964.6 ± 53.0a	4048.9 ± 75.5ab	9.79 ± 0.02a	0.997 ± 0.000a	232.9 ± 3.9ab	98.6
BF	3617.7 ± 80.8a	5296.6 ± 326.1a	4377.9 ± 330.3a	9.81 ± 0.00a	0.996 ± 0.000a	240.9 ± 1.2a	98.4
BF + OF1	3483.0 ± 14.2ab	3896.0 ± 11.3a	3975.8 ± 17.2ab	9.80 ± 0.02a	0.996 ± 0.000a	240.1 ± 10.2a	98.7
BF + OF2	3392.7 ± 9.0b	3773.4 ± 18.6a	3842.5 ± 18.6b	9.79 ± 0.01a	0.997 ± 0.000a	223.1 ± 1.2b	98.8
BF + OF3	3504.3 ± 37.8ab	3926.1 ± 24.5a	4010.7 ± 18.7ab	9.82 ± 0.03a	0.997 ± 0.000a	230.6 ± 3.9ab	98.7

CK, no fertilizer; CF, chemical fertilizers (conventional dosage for this region); BF, balanced fertilizer (30% reduction of chemical fertilizers); BF + OF1, BF + OF2, and BF + OF3, balanced fertilizer plus 3,000, 6,000, and 12,000 kg.ha^–1^ bio-organic fertilizer, respectively. Data are expressed as the mean ± standard error (SE) of three replicates. Different letters indicate significant differences at p < 0.05, according to Duncan’s test.

#### Phylum level soil bacterial abundance under different fertilization treatments

**FIGURE 3:**
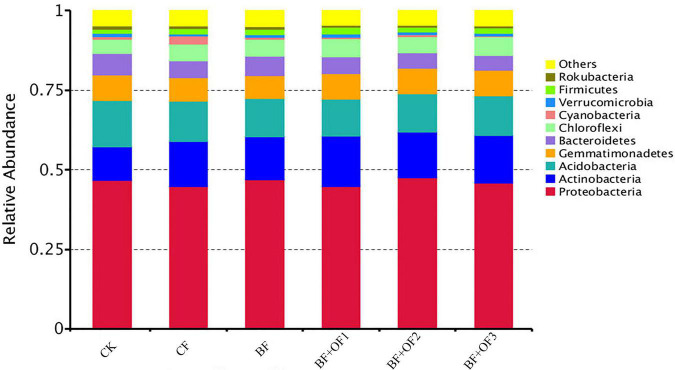
Relative abundances of species at phylum level under different fertilization treatments. CK, no fertilizer, CF, chemical fertilizers (conventional dosage for this region); BF, balanced fertilizer (70% of total chemical fertilizer); BF + OF1, BF + OF2, and BF + OF3, balanced fertilizer plus 3,000, 6,000, 12,000 kg.ha^–1^ bio-organic fertilizer, respectively.

The results show that the dominant phyla were Proteobacteria, Actinobacteria, Acidobacteria, Gemmatimonadetes, Bacteroidetes, and Chloroflexi, with average relative abundances of 45.9, 13.9, 12.7, 7.8, 5.7, and 5.2%, respectively (Figure 3). In addition, some bacterial phyla with low relative abundance were detected, including Cyanobacteria, Verrucomicrobia, Firmicutes, and Rokubacteria, with relative abundances of 0.8∼4.7%.

**FIGURE 4:**
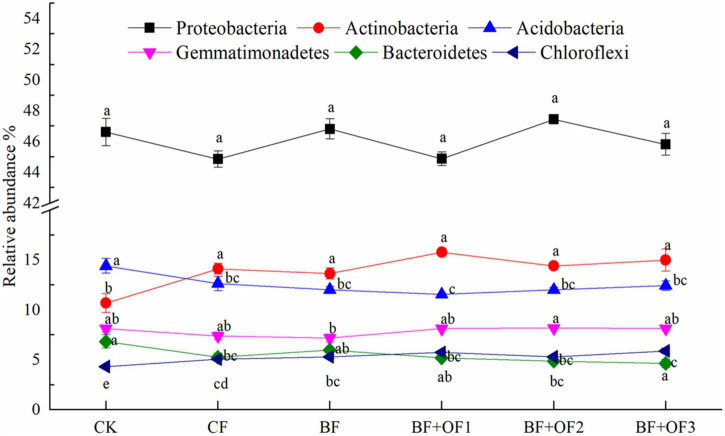
Relative abundances of six dominant bacteria at the phylum level under different fertilization treatments. CK, no fertilizer; CF, chemical fertilizers (conventional dosage for this region); BF, balanced fertilizer (70% of total chemical fertilizer); BF + OF1, BF + OF2, and BF + OF3, balanced fertilizer plus 3,000, 6,000, and 12,000 kg.ha^–1^ bio-organic fertilizer, respectively. Different letters above the bars indicate statistically significant differences by Duncan’s test (*p* < 0.05).

The effects of different fertilization treatments on the relative abundances of the six dominant bacterial phyla are shown in Figure 4. No significant differences were observed in the relative abundance of Proteobacteria among the different treatments. The relative abundance of Actinobacteria in BF decreased by 3.4% compared with that of CF, while the abundances in BF + OF1, BF + OF2, and BF + OF3 increased by 11.7, 2.0, and 6.2%, respectively. The relative abundances of Gemmatimonadetes in the BF + OF1, BF + OF2 and BF + OF3 treatments was 9.9, 10.9, and 10.3% higher than that in the CF treatment, respectively. The BF + OF3 treatment significantly improved the relative abundance of Chloroflexi compared with that in the CF treatment.

#### Genus level soil bacterial abundances under different fertilization treatments

**FIGURE 5:**
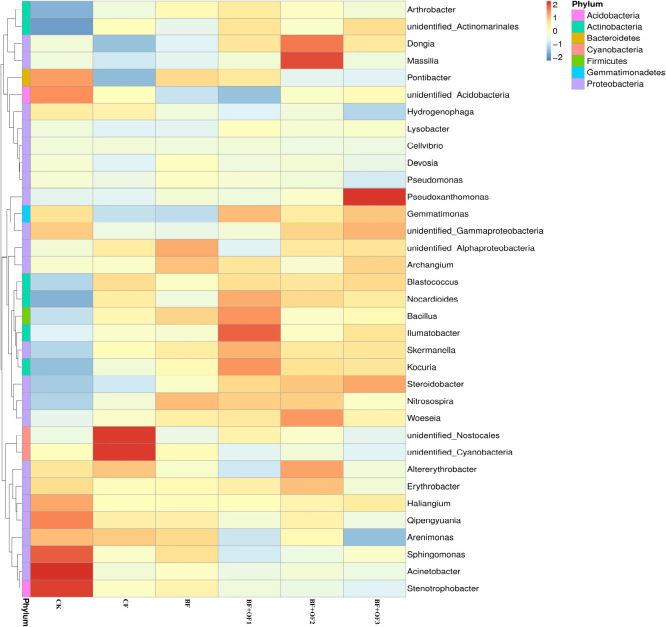
Heat map analysis of bacterial community in soil of different fertilization treatments. CK, no fertilizer, CF, chemical fertilizers (conventional dosage for this region); BF, balanced fertilizer (70% of total chemical fertilizer); BF + OF1, BF + OF2, and BF + OF3, balanced fertilizer plus 3,000, 6,000, and 12,000 kg.ha^–1^ bio-organic fertilizer, respectively. (Colors from blue to red represent that the relative abundance of the community from low to high).

The differences and similarities of species communities were verified by the color change gradients and similarities of the heat maps (Figure 5). Heat maps of the top 35 genera of soil bacteria were analyzed. Significant changes in bacterial community structure was observed in all six treatments. In the CF treatment, the relative abundances of *unidentified_Nostocales* and *unidentified_Cyanobacteria* were the highest. The application of bio-organic fertilizer enriched the soil with *Bacillus*, *llumatobacter*, *Kouria*, and *Nocardiodes* in BF + OF1 and *Massilia*, *Dongia*, *Woeseia*, and *Altereythrobacter* in BF + OF2, compared with the CF treatment. The relative abundances of *Pseudoxanthomonas*, *Gemmatimonas*, and *Nitrospirae* increased significantly in the BF + OF3 treatment compared with in the CF treatment. Moreover, the soil of the BF + OF series exhibited a more even bacterial composition than the CF soil, as shown by the color intensity.

#### Beta-diversity of soil bacteria under different fertilization treatments

**FIGURE 6:**
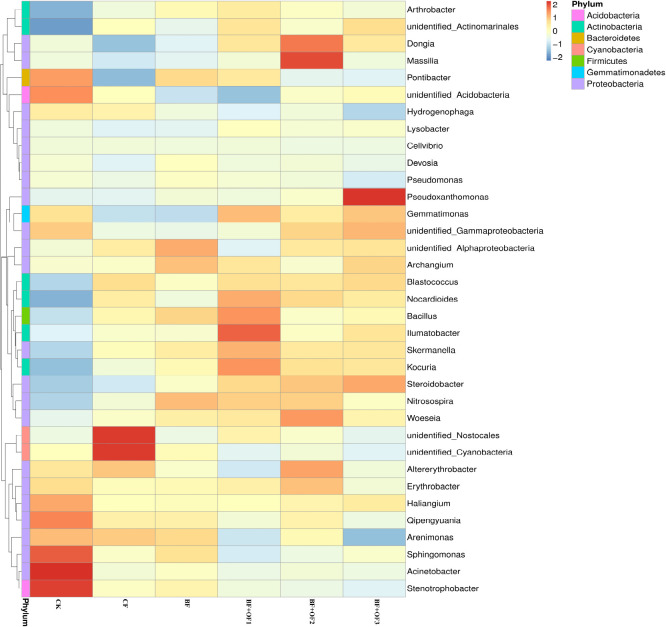
Principal component analysis (PCA) of soil under different fertilization treatments based on the operational taxonomic unit (OTU) level. CK, no fertilizer, CF: chemical fertilizers (conventional dosage for this region); BF, balanced fertilizer (70% of total chemical fertilizer); BF + OF1, BF + OF2, and BF + OF3, balanced fertilizer plus 3,000, 6,000, and 12,000 kg.ha^–1^ bio-organic fertilizer, respectively.

Figure 6 shows that, principal components 1 and 2 explain 9.3 and 7.7% of the variance of variables, respectively, and the cumulative variance contribution rate is 17.0%. The samples were divided into two groups (Figure 6). The treatments with no fertilizer and only chemical fertilizer (CK, CF, and BF) clustered more closely together.

In contrast, BF + OF1, BF + OF2, and BF + OF3, in which chemical fertilizer reduction was based on balanced fertilization combined with biological organic fertilizers, formed one group. There was an obvious difference between the two groups, showing a good dispersion pattern.

#### Effects of environmental factors on soil bacterial community distribution under different fertilization treatments

**FIGURE 7:**
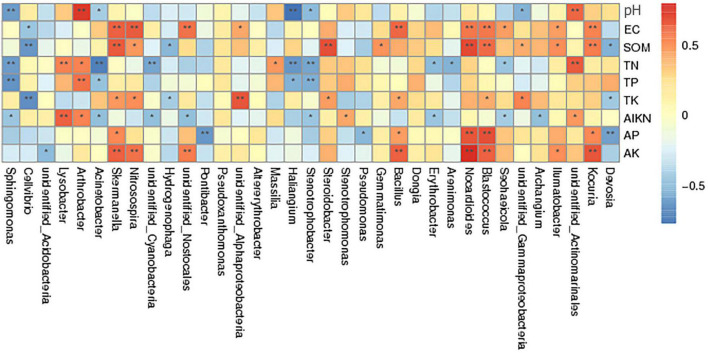
Spearman’s correlation analysis of soil bacterial community and soil physicochemical properties under different treatments. CK, no fertilizer; CF, chemical fertilizers (conventional dosage for this region); BF, balanced fertilizer (70% of total chemical fertilizer); BF + OF1, BF + OF2, and BF + OF3, balanced fertilizer plus 3,000, 6,000, and 12,000 kg.ha^–1^ bio-organic fertilizer, respectively. **Correlation is significant at the 0.01 level; *correlation is significant at the 0.05 level.

Correlation analysis of different bacterial genera and soil physicochemical properties showed that SOM was positively correlated with the abundances of *Blastococcus*, *Kocuria*, *Nocardioides*, *Steroidobacter*, and *Skermanella* (Figure 7). Soil alkaline nitrogen, AK, and AP contents were positively correlated with the abundances of *Kocuria*, *Nocardioides*, *Skermanella*, *Nitrosospira*, *Blastococcus*, *Lysobacter*, and *Bacillus*. The abundances of these bacteria were significantly higher in the chemical fertilizer reduction combined with biological organic fertilization treatments (BF + OF1, BF + OF2, and BF + OF3) than in the conventional chemical fertilization (CF) treatment. In contrast, the abundances of *Stenotrophobacter*, *Haliangium*, *Acinetobacter*, *Cellvibrio*, and *Sphingomonas* was negatively correlated with TN, TP, SOM, and PH.

## Discussion

It is generally considered a practical and reasonable practice to apply both chemical and organic fertilizers to maintain the sustainable development of agricultural ecosystems (Gentile et al., 2008). In our study, we found that reducing chemical fertilizers in conjunction with bio-organic fertilizer use could improve SOM, nutrient availability, enzyme activity, bacterial community diversity and richness, and crop yield.

Changes in soil physicochemical properties were closely linked to application of organic fertilizers. The SOM, AN, AP, and AK in the soil increased after using the bio-organic fertilizer. Similarly, Baldi et al. (2016) reported that SOM and TN contents of orchard soils increased with organic fertilizer application. Zhao et al. (2016) found that the combined application of organic and inorganic fertilizers could improve the availability of soil nutrients and increase crop economic yields to a certain extent. Furthermore, Organic fertilizer can improve saline soil by improving soil aggregate structure, soil bulk density, soil permeability and alkalization (Li-ping et al., 2015). The soil pH and EC values of the BF + OF2 and BF + OF3 treatments were significantly lower than those of CF treatment, which is similar to the findings of Jiang et al. (2019). This indicated that adding appropriate amount of bio-organic fertilizer can maintain the stability of soil pH and prevent the partial acid or alkali of soil from affecting the growth and development of crops. According to Chang et al. (2007) there may be a reason why a high organic matter content can diminish the harmful effects of soluble salts on plant growth. Soil enzymes play an essential role in regulating soil nutrient turnover and are derived primarily from soil microorganisms (Yang et al., 2008). These enzymes are involved in the transformation of soil C, N, and P, such as sucrase, urease, alkaline phosphatase, and β-glucosidase. Using bio-organic fertilizer can improve soil enzyme activity and promote nutrient cycling, a finding that is in agreement with that published elsewhere (Zhao et al., 2016).

Adopting bio-organic fertilizers effectively improves soil environmental conditions and the soil microbiome (Tao et al., 2015). Therefore, soil microflora play a central role in decomposing loaded organic matter and cycling nutrients, particularly the most abundant group of bacteria, which are essential for ecological services (Zhang et al., 2012). Ding et al. (2016) reported that soil microbial diversity increased by combining organic and inorganic fertilizers, while the Shannon, Chao1, and Simpson indices of inorganic fertilizers with manure (MNPK) treatment significantly exceeded those of nitrogen, phosphorus and potassium inorganic fertilizer (NPK). We found that chemical fertilizer reduction combined with bio-organic fertilizers significantly increased soil bacterial diversity and richness compared to with conventional fertilization.

Different fertilization methods affect microbial community composition and structure. According to PCA analysis (Figure 5), the bacterial community composition were distinguished by different fertilization regimes. As confirmed by previous studies (Gu et al., 2019; Jiang et al., 2019; Brenzinger et al., 2021), the soil bacterial community structure changed after organic fertilizer application. In this study, the bio-organic fertilizer combination treatments increased the relative abundances of beneficial bacteria, such as Proteobacteria, Actinobacteria, Gemmatimonadetes, and Chloroflexi. Proteobacteria are thought to play an essential role in soil carbon, nitrogen and sulfur cycling (Rampelotto et al., 2013), and also have a strong resistance to heavily contaminated soils, especially under heavy metal stress (Burkhardt et al., 2011; Li et al., 2016). Actinobacteria play a critical role in the decomposition of organic matter and are highly resistant to UV radiation, heat, and desiccation (Essoussi et al., 2012; Hamedi et al., 2013). Gemmatimonadetes can promote the accumulation of P in the soil and root growth (Zhang et al., 2003). Chloroflexi can generate energy through photosynthesis and promote carbon conversion and utilization under conditions of low nutrient availability (Fullerton and Moyer, 2016). As with most fertilization experiments, these results are consistent. For example, the application of bio-organic fertilizer increased the abundances of Actinobacteria, Bacteroidetes, and Chloroflexi, and these bacteria suppress bacterial wilt in tomatoes (Zheng et al., 2020). With the addition of organic fertilizer, the microbial community composition was altered, leading to an increase in Proteobacteria, Bacteroidetes, and Gemmatimonadetes (Zhang J. et al., 2019). Higher relative abundances of *Massilia*, *Dongia*, *Pseudoxanthomonas*, *Gemmatimonas*, and *Nitrospirae* was observed at the genus level in the BF + OF1, BF + OF2, and BF + OF3 treatments compared with the CF treatment (Figure 4). The genus *Massilia* has been frequently reported as a rhizosphere and endorhizal colonizer. Furthermore, *Massilia* was reported to promote plant growth by producing auxin and siderophore, and by antagonizing the growth of Phytophthora infestans *in vitro* (Ofek et al., 2012). The genus *Dongia* is frequently reported as a beneficial microbe that suppresses the growth of soil-borne pathogens (Han et al., 2018). At present, the severity of soil and air pollution are increased by the extensive use of fertilizers and pesticides (Jianmin et al., 2007). Bioremediation is the most powerful and influential method of decontaminating polluted ecosystems. As confirmed by previous studies, the genus *Pseudoxanthomonas* functions in phenanthrene degradation and pesticide residue degradation (Patel et al., 2012; Talwar and Ninnekar, 2015). Wu et al. (2016) reported that *Gemmatimonas* was beneficial for bacteria wilt control. *Nitrospirae* can promote soil nutrient cycles and effectively reduce the salinity and alkalinity of soil to improve soil fertility (Zhang Y. et al., 2019; Xu et al., 2021). The increase of beneficial microorganisms in soil can promote plant growth and increase yield (Srinivasarao and Manjunath, 2017; Brenzinger et al., 2021). Moreover, Pseudomonas fluorescens can produce plant hormones and iron carriers (iron chelators) that promote growth, and also help to dissolve minerals in soil (Doni et al., 2019). In summary, chemical fertilizer reduction combined with bio-organic fertilizers enhances the structure and function of soil microorganism communities. As a result, beneficial bacteria are more abundant, which contributes to soil nutrient and yield improvement.

The soil environment plays a critical role in influencing the structure of microbial communities, as evidenced by numerous studies. For example, Jin et al. (2021) observed that AP, TN, SOM, EC, and pH were the primary drivers of microbial community structure, which is consistent with the results of this study. Redundancy analysis (RDA) revealed that organic matter (OM) and available phosphorus (AP) were the main factors driving the bacterial and fungal community structure (Ji et al., 2021). Zheng et al. (2022) results suggest a strong link between microbial community traits and SOC accumulation rate. Chemical fertilizer reduction combined with organic fertilizer and straw can effectively improve soil fertility and nutrient availability, altered the community structure of both bacteria and fungi (Shen et al., 2018; Tripathi et al., 2018; Wang et al., 2019). The redundancy analysis (RDA) results showed that the structure of the bacterial community was significantly correlated with SOM and TN, and the fungal community structure was strongly correlated with AP, SOM, and TN (Wu et al., 2021). Soil pH is one of the main factors affecting soil chemical, physical and biological properties (Buckman and Brady, 2002). The pH value in soil can be decreased due to inappropriate fertilization patterns through long-term fertilization experiments (Cai et al., 2015). Previous studies have proved that soil pH changes are closely related to soil microbial changes. The composition of soil bacterial community is significantly affected by soil pH (Shen et al., 2018; Tripathi et al., 2018). Xiong et al. (2012) believe that pH is the most decisive factor determining the structure of soil microbiomes. Soil pH is more important than nutrients in shaping bacterial communities in agricultural soils, including their ecological functions and biogeographic distribution (Wang et al., 2019). This may be because the fertilizer regime is one of the most influential determinants of soil environmental change and further affects the composition of the soil microbial community. Organic fertilization is the most efficient approach for increasing SOM content and improving soil fertility (Lin et al., 2019). Qiao et al. (2019) found that combined organic and bio-organic fertilizers provide more available nutrients, which is advantageous to crop growth and development. Bio-organic fertilizer application significantly affected the bacterial community in the cauliflower cropping soil. The relative abundances of *Blastococcus*, *Kocuria*, *Nocardioides*, *Nitrospirae*, and *Skermanella* bacteria were positively correlated with SOM content. The *Blastococcus* genus can degrade organic material through proteolysis (Duan et al., 2019). These results showed that the addition of microbial fertilizer could increase the content of organic matter and the amounts of beneficial bacteria in soil, then promote the absorption and utilization of crop nutrients. *Kocuria* abundance was significantly correlated with soil EC, which may be due to its higher salt tolerance during growth (Takarada et al., 2008). After the application of bio-organic fertilizer, the relative abundance of Bacillus was positively related to AK, owing to the ability of Bacillus to dissolve K (Tahir et al., 2018; Pramanik et al., 2019).

## Conclusion

Chemical fertilizer reduction combined with appropriate bio-organic fertilizers (6,000 kg.ha^–1^ and 12,000 kg.ha^–1^) can increase cauliflower yield by improving soil fertility, promoting soil enzyme activity, altering microbial community composition, and stimulating potentially beneficial microbial functions. The fertilization pattern is adequate for the sustainable development of cauliflower production and soil environment protection in the northwest plateau region of China.

## Data availability statement

The datasets presented in this study can be found in online repositories. The names of the repository/repositories and accession number(s) can be found in the article/supplementary material.

## Author contributions

XX and JLi designed the study and wrote the manuscript. XX, JLi, CG, HY, and ZF performed the work. JLi and LJ analyzed the data. JY, JLy, and GZ revised the manuscript and provided resources. All authors contributed to the article and approved the submitted version.

## Conflict of interest

The authors declare that the research was conducted in the absence of any commercial or financial relationships that could be construed as a potential conflict of interest.

## Publisher’s note

All claims expressed in this article are solely those of the authors and do not necessarily represent those of their affiliated organizations, or those of the publisher, the editors and the reviewers. Any product that may be evaluated in this article, or claim that may be made by its manufacturer, is not guaranteed or endorsed by the publisher.

## Footnotes

1
http://ccb.jhu.edu/software/FLASH/

2
http://qiime.org/scripts/split_libraries_fastq.html

3
https://github.Com/torognes/vsearch/

4
http://www.drive5.com/uparse/

5
http://www.arb-silva.de/
